# KEY DIMENSIONS OF OASIS, AN OLDER-ADULT DRIVEN MODEL OF AGING-IN-PLACE IN CANADA

**DOI:** 10.1093/geroni/igz038.3477

**Published:** 2019-11-08

**Authors:** Vincent DePaul, Catherine Donnelly, Simone Parniak, Oasis Study Collaborative

**Affiliations:** 1 Queen's University, Kingston, Ontario, Canada; 2 Oasis Study Collaboration, Ontario, Canada

## Abstract

Oasis Senior Supportive Living (Oasis) is an active aging-in-place model created by older adults in a naturally occurring retirement community, such as an apartment building. The program is member-driven so that participating older residents determine the programming and services that best address their needs. The first Oasis program was established in an apartment building in Kingston, Canada and has been running for more than ten years. Preliminary evaluations of the Oasis program demonstrate that its members report feeling more socially connected, are more physically active, and have increased nutrition as a result of participation. In-depth interviews were conducted with Oasis members and key program stakeholders to identify the core dimensions of the Oasis program that has led to its success in supporting active aging in place. Interviews were audio-recorded and transcribed verbatim. Thematic analysis was used to identify, analyze and report themes. Four themes emerged: (1) nutrition, social and physical activities as critical programming pillars; (2) the importance of active member participation and decision-making; (3) the need for onsite support to facilitate programming; and (4) Oasis as a family. These findings highlight the need for programming that is designed for and by older adults. Supporting older adults to come together and form community is key to healthy and active aging. Identification of these elements is critical to modelling Oasis in other community contexts. Oasis is currently being expanded to seven new communities across Ontario using a participatory action research approach.

